# Dataset for flood area recognition with semantic segmentation

**DOI:** 10.1016/j.dib.2023.109768

**Published:** 2023-11-04

**Authors:** Naili Suri Intizhami, Eka Qadri Nuranti, Nur Inaya Bahar

**Affiliations:** Institut Teknologi Bacharuddin Jusuf Habibie, Indonesia

**Keywords:** Flood, Semantic segmentation, Video, Object detection, Image

## Abstract

Floods are natural disasters that repeatedly occur in Indonesia, causing substantial material losses and claiming many lives. Meanwhile, social media data has emerged as a valuable resource for analyzing user behaviour and interests, and its use for flood-related information is increasing. In this paper, we present a flood dataset collected from Instagram Reels, which consists of videos depicting flood events in Parepare. Every video was collected from different areas, time conditions and viewpoint, and converted into image form. The data set includes 7248 images. Images undergo preprocessing to ensure a clear depiction and differentiation of the flood event from the surrounding elements. Annotations given to each object, using a different color label, facilitate recognition and understanding of various computer vision applications. Overall, this flood dataset is a valuable resource for computer vision research, especially semantic segmentation method and promotes the development of algorithms for flood area identification and object recognition in flood-affected areas.

Specifications Table

**Table:** 

Subject	Computer Vision and Pattern Recognition
Specific subject area	Dataset for flood area recognition and object detection with semantic segmentation
Data format	Raw, Filtered, and Segmentated
Type of data	Image and Video
Data collection	We collected this data from social media Instagram. The collected data consists of video data showing flood events. The original video is over a minute long. Then the video is transformed into images. Next, annotate the images with appropriate labels for each object (floods, buildings, people, vehicles, sky, and plants). This annotation process is done manually by a group of annotators using tools for object annotations. Because the images were taken from different regions and at other times, there may be differences in the lighting, layout, or scale of objects in the image frame.
Data source location	Institut Teknologi Bacharuddin Jusuf Habibie, Parepare City, South Sulawesi, Indonesia
Data accessibility	Repository name: Flood Amateur Video for Semantic Segmentation Dataset [1] Data identification number: 10.17632/3kzr8mt8s2.3 Direct URL to data**:**https://data.mendeley.com/datasets/3kzr8mt8s2/3

## 1 Value of the Data

• The dataset comprises 21 videos and 7248 images divided into raw images, and annotated images. The annotated image consists of six different classes, each differentiated by color.
• The dataset helps increase the accuracy of machine learning or deep learning models in flood recognition and object detection using the semantic segmentation method.
• The flood dataset includes a variety of time conditions and viewpoints that can help to improve the accuracy of machine learning models.
• The dataset is useful for training, testing, and validating flood area recognition and object detection in images or videos.
• Researchers can use this dataset in images/videos for object detection, and semantic segmentation methods.
• The dataset will help build applications for intelligent flood disaster preparedness systems, and researchers can also use this dataset to develop better flood recognition models and methods in the future.

## 2 Data Description

Flood is one of the disasters that often occurs not only in Indonesia [2,3], but also worldwide [4]. Floods are natural disasters where rivers, lakes, or dams cannot accommodate large amounts of water from high rainfall, so it overflows and submerges the land [2,5]. The flood disaster had a negative impact in the form of material losses and claimed a large number of lives [3].

**Fig. 1:**
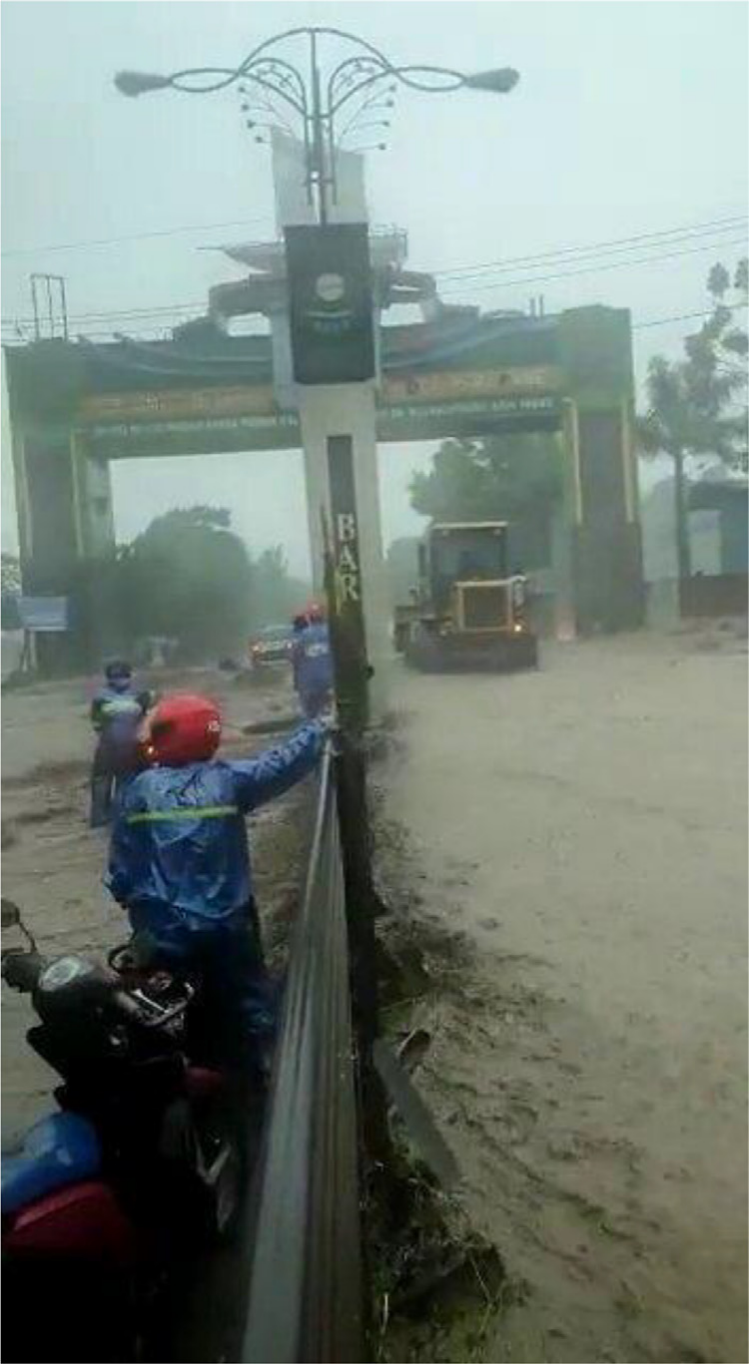
Image of flood video.

Using data from social media is one of the methods used to analyze a user’s behavior, interest, and tendencies [6]. The development of obtaining information from social media continues to increase. Users worldwide can access information on social media for flood disaster cases, making it the most reliable and fastest source. Fig. 1 shows the image sample of the flood video.

The flood dataset consists of three folders: the video folders, the images folder, and the annotation folder. A total of 21 videos in the video folder show the flood incident in the city of Parepare collected from @ParepareInformasi Instagram account. We collect video data manually without using web scraping and with the permission of the account owner. Each video has a different size due to differences in quality from each user who uploaded it. This variation provides diversity within the dataset and reflects other conditions from various video sources. In the image folder, there are 3754 images resulting from splitting the video into several frames. The size of each image follows the size of the original video. By not equating the size of each image, this dataset reflects the different size and aspect ratio variations in the flood events in the city of Parepare. We have annotated 3494 images in the annotation folder. This annotation folder becomes important information in training the model for object recognition and semantic segmentation.

The flood dataset includes a variety of time conditions and viewpoints. The time conditions of day can have a significant impact on the appearance of a flood. Floodwater is often darker and difficult to recognise at night, which can make it difficult to distinguish floodwater and other objects. Additionally, the amount of sunlight can affect the visibility of floodwater and other objects. The viewpoint from which a flood is photographed can also affect the information that is captured. For example, a photograph taken from a bird's-eye view can provide a wider view of the extent of the flooding, while a photograph taken from ground level can provide a more detailed view of the damage caused by the flooding. The time conditions and viewpoints in a flood dataset are differences of this dataset has compared to other datasets and can be useful for researchers to improve the accuracy of their deep learning models.

The time conditions and viewpoints in the flood dataset are differences that this dataset has compared to other datasets and can be useful for researchers to improve the accuracy of their machine learning models. The time conditions and viewpoints in a flood dataset is can be useful for researchers to improve the accuracy of their machine learning models.

## 3 Experimental Design, Materials and Methods

**Fig. 2:**
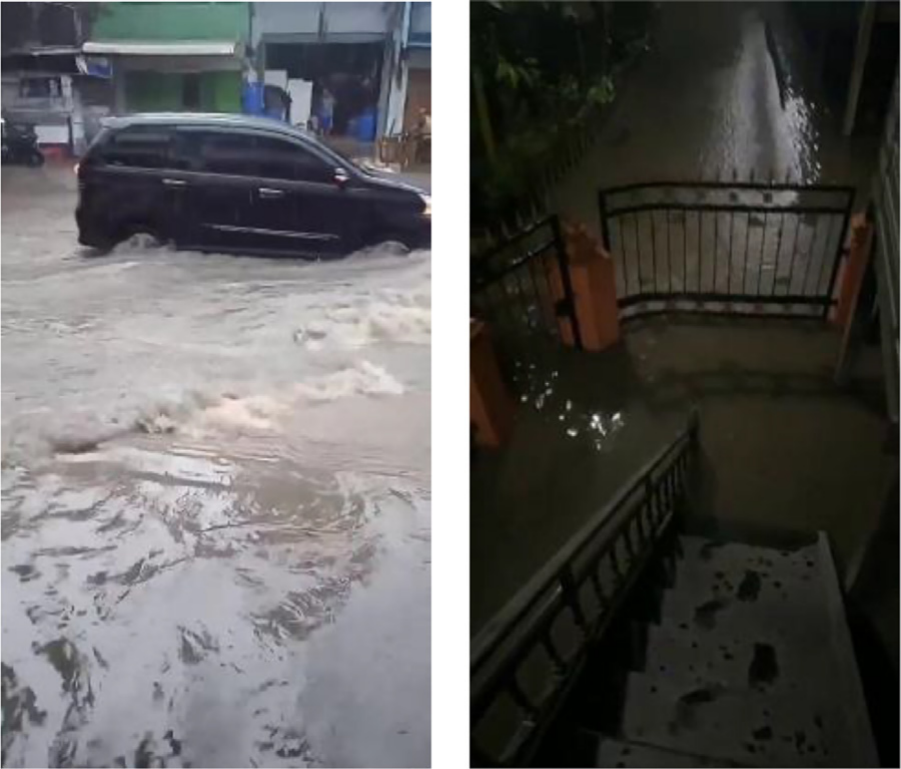
Images sample of flood taken from the front (left) and from above (right).

Several criteria form the basis for video data retrieval. We implement these criteria to ensure that the created dataset improves accuracy in identifying flood areas and objects around them. Among other things, we base the criteria for selecting a flood disaster video on the location of the incident within the Parepare City area. The video's quality, which will be used as a dataset, must be good enough to display flood events clearly without being blurry. Additionally, we require the video to be taken from the front (horizontal) and from above (vertical). Fig. 2 shows images of the flood taken from different viewpoints and time conditions.

**Fig. 3:**
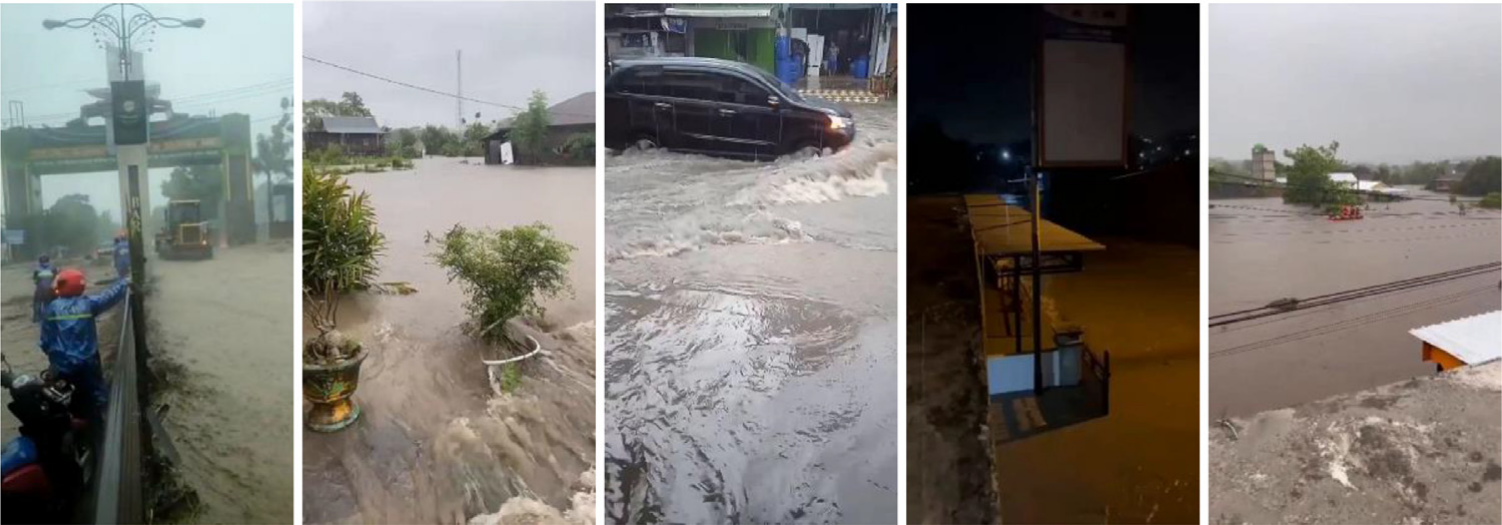
Image samples that have passed the preprocessing stage.

After successfully splitting the entire video into individual images. The next step involves data preprocessing. This stage aims to filter and select images that will form the dataset. Specific criteria must be met for an image to be included in the dataset, such as a clear depiction of a flood event without blurriness and distinct differentiation between the flood and the surrounding objects. As a result of this preprocessing stage, 3754 images are generated and ready for dataset use and we call them raw images. Fig. 3 displays a sample of images that have successfully passed through the preprocessing stage.

Furthermore, we proceed to annotate the images. Annotation involves assigning different colors to each object within an image. This process enables more straightforward distinction and recognition of various elements present in the image. The objects in the image are categorized into different classes and are distinguished by color labels [7,8]. For example, floods are marked in dark blue, buildings in red, plants in green, people in sage green, sky in light blue, and vehicles in orange.

Computer Vision Annotation Tool (CVAT) is a web-based software tool designed for the purpose of data annotation. CVAT can assist with three primary machine learning tasks that need image data: object recognition, image classification, and segmentation. With CVAT, users can annotate image or video data for each of these machine learning tasks. The software's user-friendliness is attributed to its uncomplicated interface, which presents only the essential menus. The features are readily comprehensible and user-friendly. Moreover, there are numerous tutorials available on the internet for learning how to use CVAT. So we made a decision to use the CVAT application (https://www.cvat.ai/) for this annotation process.

**Fig. 4:**
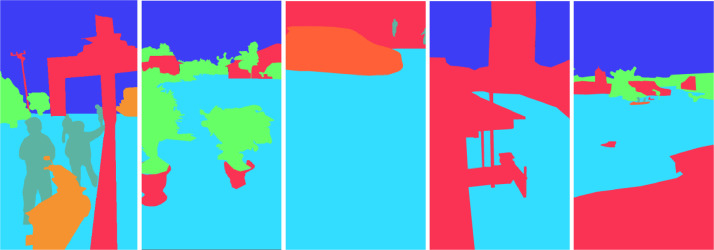
Images sample annotating with a color label on each object.

Using CVAT, we developed an annotation guide to assist annotations during the picture annotation process. The annotation processes are illustrated with sample photographs in the annotation guide. In addition, in order to help annotators better understand how to annotate photos in accordance with their needs, the annotation guide offers instances of improper or inappropriate annotations. Fig. 4 shows the image sample annotating with a color label on each object.

Based on these criteria, this study collected 21 videos at different locations. In subsequent data collection activities, we will transform videos from Instagram social media into images. Tabel 1 presents the number of row and annotation images obtained from each video and the information about time condition and viewpoints.

**Tabel 1:** Video information and the number of images obtained from each video.

Video title	Time_conditions (darkness or light)	Viewpoints (horizontal or vertical)	Number of annotation images	Number of raw Images
2022111801	l	h	300	300
2022111802	l	h	134	134
2022111803	l	h	200	251
2022111804	l	h	108	108
2022111805	l	v	300	300
2022111806	l	h	188	188
2022112001	l	h	299	299
2023020101	d	h	173	173
2023020102	d	h	101	101
2023020103	d	h	95	95
2023020104	d	v	121	121
2023020105	d	h	212	212
2023020106	d	h	77	77
2023020107	d	h	175	175
2023020108	d	h	110	110
2023020201	d	h	20	220
2023020202	d	h	130	130
2023032401	d	h	271	271
2023033001	d	v	120	120
2023033002	d	h	215	222
2023033003	d	h	152	152
**Total number of images**			3494	3754

Challenges encountered in collecting data from social media include things like inconsistent video or image quality, which greatly depends on the capability of the user's mobile phone camera, as well as the unprofessional position to shoot. This is because the bulk of users primarily focus on capturing flood situations without prioritizing photograph quality. Furthermore, acquiring consent from the account proprietor poses an additional challenge.

These annotations enable the system to recognize and comprehend the components present in the image, facilitating various computer vision and image processing applications. Flood datasets can be developed using applications other than CVAT. Alternatively, it may utilize alternative apps of similar functionality or photo editing applications. This dataset contains image data in the JPG/JPEG format for raw images and the PNG format for annotated images. All images can be accessed and viewed using an image reader application.

## Limitations

The limitation of the geographical focus that we use is around our institution, namely Institut Teknologi Bacharuddin Jusuf Habibie which is in the city of Parepare.

## Ethics Statement

The dataset collected only focuses on flooding in five areas of Parepare City, South Sulawesi. Videos are collected from @ParepareInformasi Instagram accounts and with the permission of the account owner. We collect video data manually without using web scraping. All videos and images are anonymized so our dataset does not share any identifiable information.

## CRediT authorship contribution statement

**Naili Suri Intizhami:** Methodology, Conceptualization, Investigation, Validation, Writing – original draft, Writing – review & editing, Supervision, Visualization, Data curation. **Eka Qadri Nuranti:** Software, Investigation, Validation, Resources, Data curation, Writing – original draft, Writing – review & editing. **Nur Inaya Bahar:** Software, Resources, Data curation.

## Data Availability

Flood Amateur Video for Semantic Segmentation Dataset (Original data) (Mendeley Data)

## Appendix Supplementary materials

Image, application 1

Image, application 2
