# Supplementary material for: Dataset for flood area recognition with semantic segmentation
Source: Data Brief. 2023 Nov 4;51:109768. doi: 10.1016/j.dib.2023.109768 (PMC10661843; doi:10.1016/j.dib.2023.109768)
Supplement: Supplementary file 1 [file mmc1.pdf]

Respected Copyright Holder,

I am writing to inform you that the "@PareparеInformasi" instagram account contains a flood disaster video for which you hold the copyright.

Institut Teknologi Bacharuddin Jusuf Habibie intends to use some flood video content for a computer vision aspect of flood disasters in a research report conducted by Institut Teknologi Bacharuddin Jusuf Habibie lecturers and students, with the account to be published in a journal.

If you agree to permit us, kindly sign this permission request letter and return a copy via email.

We sincerely appreciate your consideration of our permission request.

Truly,

Naili Suri Intizhami

Permit Officer Admin @PareparеInformasi

By signing below, I warrant that I have permission to grant the permissions requested in this letter, and I present you with such permission.

Signature :

Date : August 4, 2023

Dipindai dengan CamScanner

Permit Officer Admin @PareparelInformasi

By signing below, I warrant that I have permission to grant the permissions requested in this letter, and I present you (Naili Suri Intizhami) with such permission.

Signature :

Date : August 4, 2023

Dipindai dengan CamScanner
