# Supplementary material for: Dataset for flood area recognition with semantic segmentation
Source: Data Brief. 2023 Nov 4;51:109768. doi: 10.1016/j.dib.2023.109768 (PMC10661843; doi:10.1016/j.dib.2023.109768)
Supplement: Supplementary file 2 [file mmc2.pdf]

Guidelines for Flood Dataset Annotation for Semantic Segmentation Method using CVAT

Data annotation is the process of adding information (metadata) to data to make it more understandable and beneficial for machine learning algorithms. This information/metadata can include labels, object classes, or descriptions that can be used to identify objects, classify images, or provide context for analysis.

The annotated data type consists of flood images, using semantic segmentation annotation. Semantic segmentation is the process of assigning class labels to every object within an image. Therefore, it can be said that every object within the image has a different object class. For example, in an image containing the sky and buildings, using the semantic segmentation method, these two objects will be assigned different color labels.

CVAT is an application tool that can be used for data annotation. This application is primarily designed for annotating images and videos. It is user-friendly due to its straightforward interface, displaying only the necessary menus. Its features are easy to understand and use. Additionally, CVAT provides shortcut information for each feature, such as the 'F' shortcut for the 'next' feature. Furthermore, there are numerous tutorials available on the internet for learning how to use CVAT.

In the author's case, the images will be annotated using the semantic segmentation method. It has been previously mentioned that the annotated images are flood event images. The author not only focuses on flood objects but also assigns labels to several other objects. Each image will be annotated, categorizing each object in the image into six different classes, as shown in the table below:

Colour Class (Hex)	Description
66FF66	Plants (Trees, flowers, and etc)
FA3253	Buildings (Buildings, fences, wood, bridges, and etc
FF6037	Vehicles (Cars, motorcycles, bicycles, and etc)
33DDFF	Flood (Standing water, brown water, and etc)
3D3DF5	Sky (Morning, daytime, evening, and night sky, and etc)
5CB3A2	People (People standing, walking, using umbrellas, and etc)

Image Annotation Steps using CVAT:

1. Create a new project, name the project, and define class labels for each object (annotation method: semantic segmentation).
2. Create a new task, name the task, and select the previously created project, choosing the project will adopt the class labels from that project.
3. Upload the images to be annotated. CVAT allows users to upload multiple images at once.
4. Klik tombol submit & open, setelah itu pengguna akan diarahkan kembali ke daftar task yang telah dibuat.

5. Click 'Open' on the previously created task, and select 'Job' to view the uploaded images.
6. Annotate each object by selecting the polygon feature and specifying the object label you want to annotate.
7. You need to select the first object to be annotated. Determine the farthest or innermost object. In the example image, the sky object is the farthest, so it will be annotated first.

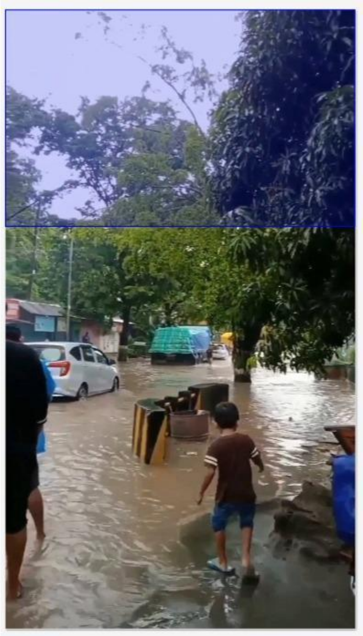

   Since the sky is the first object, a rectangular shape is sufficient.

8. After annotating all the first objects, determine the second innermost object in the image. In the example image, the second innermost object is a tree. To annotate the second object without being distracted by the first object, press the 'switch hidden property' or move the cursor towards the first object and press 'H'. This is done to temporarily hide the shape of the annotation for the first object. Then, begin annotating the tree object.

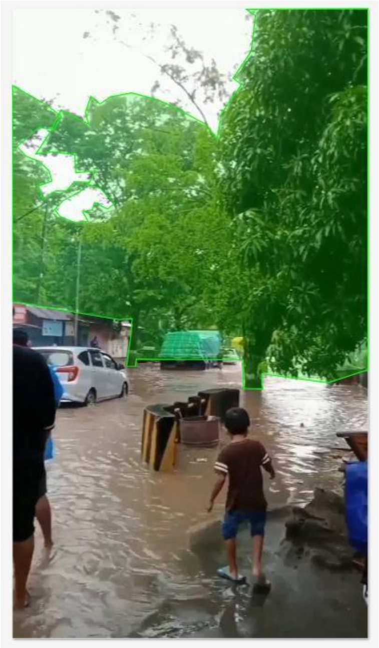

Please remember, at this early stage, the tree object does not require detailed annotation, especially if the tree object will intersect with other objects that have not been annotated.

9. After annotating the second object, proceed to determine the third innermost object. In the example image, the third object is a building. Before annotating the third object, follow the same process in step number eight. After that, begin annotating the building object.

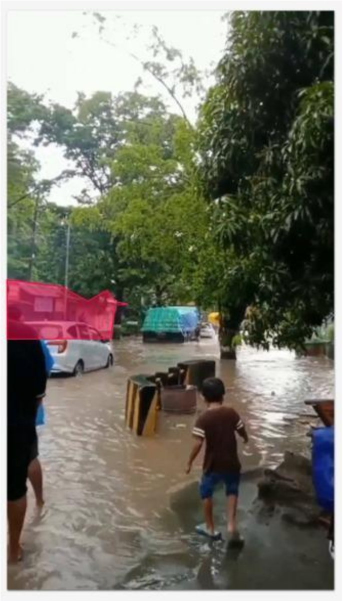

10. After completing the third object, proceed to determine the fourth object to be annotated. In the example, the flood object is the fourth object because vehicles require many polygon points and are located among many objects.

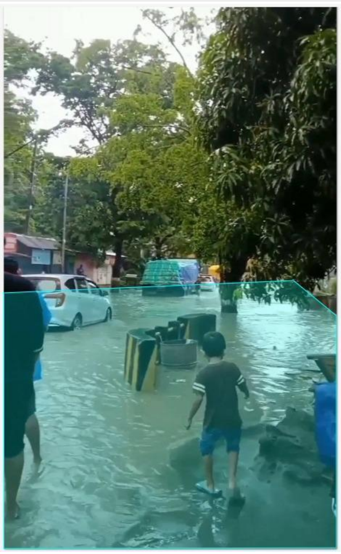

It is important to note that there are areas of the tree object affected by the annotation of the flood object, so corrections need to be made to the tree object's points.

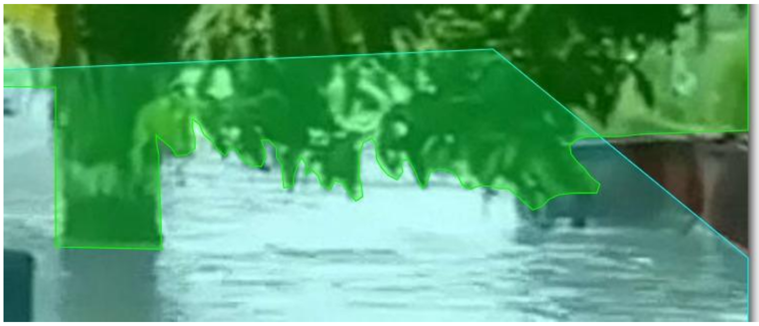

11. After completing the fourth object, proceed to determine the fifth object. In the example image, the fifth object is a vehicle.

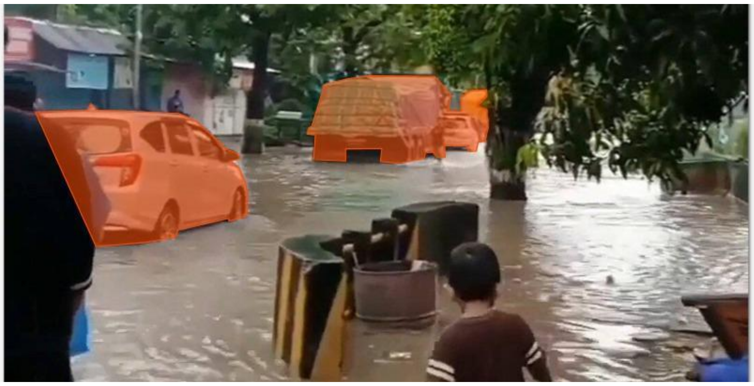

12. After completing the fifth object, there is a bridge object identified as a building that hasn't been annotated. Therefore, the sixth object is the bridge and any other objects that can be identified as buildings.

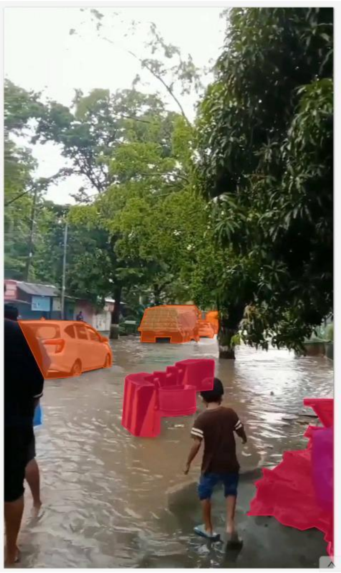

13. Then, the last object to be annotated is people. In the example image, there are 4 people visible. Use a polygon shape to annotate the people objects.

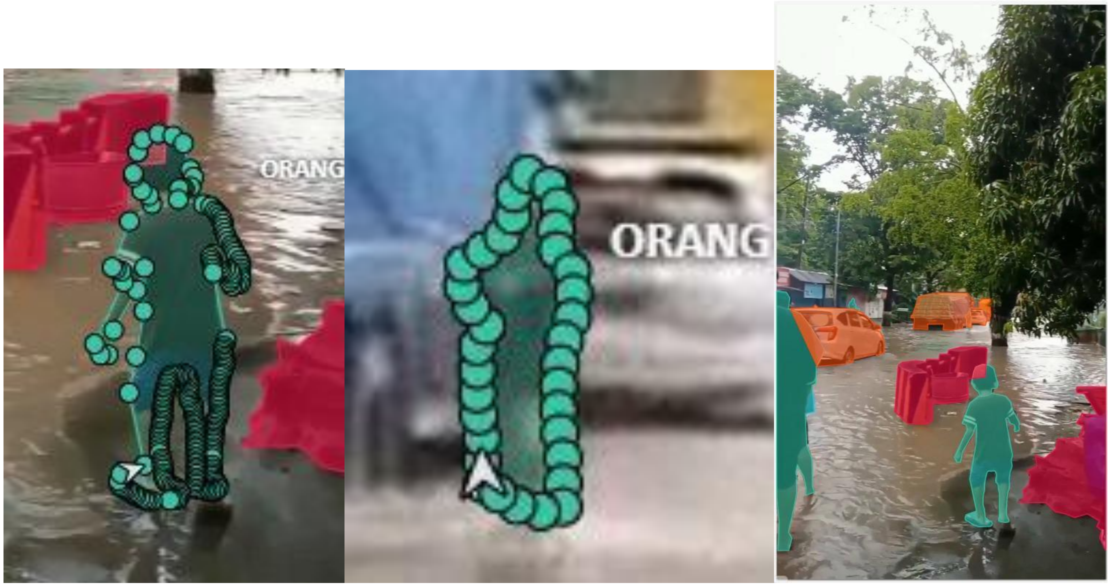

14. Save the completed work by pressing the save button.
15. Click the 'go next' icon or press 'F' to continue the annotation work to the next image.

16. If all the images have been annotated, export the data by moving the cursor to the menu. Then, an option to export the job dataset will appear. Once selected, the export data format will be displayed. Please choose 'segmentation mask 1.1' to export the dataset in the semantic segmentation format.
17. Wait for the download process to finish, then unzip the dataset to be able to use the exported dataset. An example of the successfully exported images is shown below.

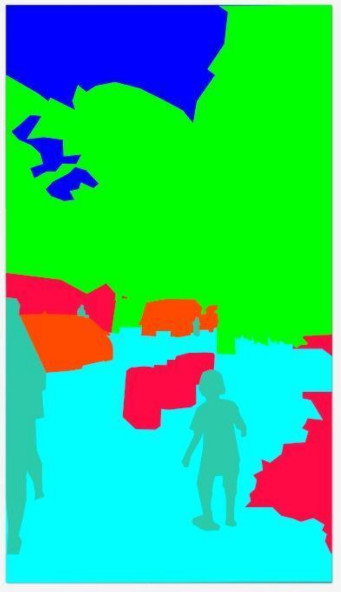

Here are some samples of incorrectly or erroneously tagged images and also examples of correctly-annotated images to help you better understand image annotations that meet the requirements of this study.

No	Raw Image	Inaccurately annotated images	Accurately annotated images	Information
1.	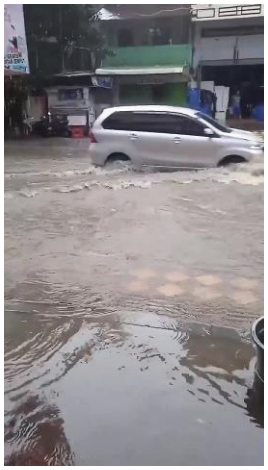	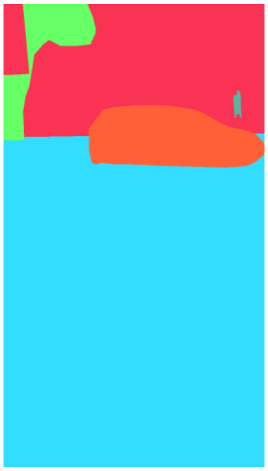	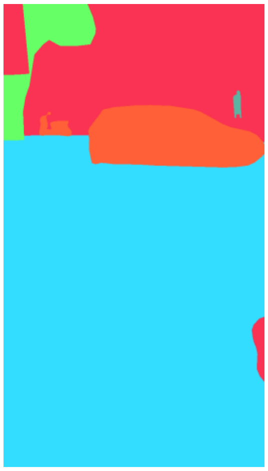	Some object are identified as floods, but they are not actually floods

2	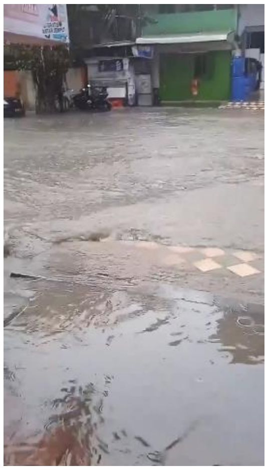	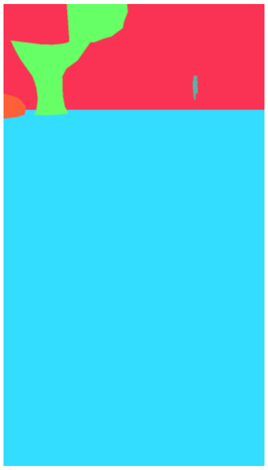	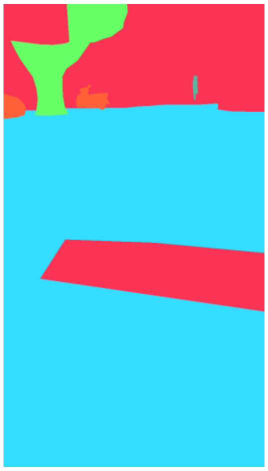	Some object are identified as floods, but they are not actually floods
3	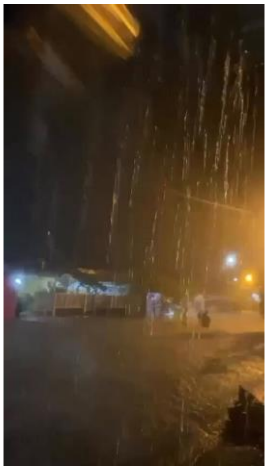	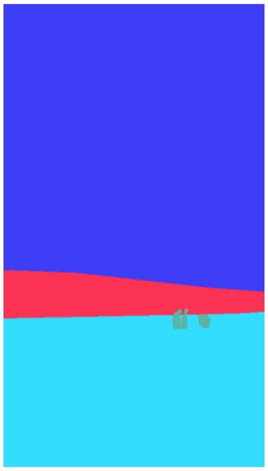	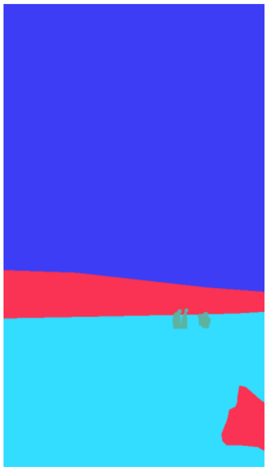	Some object are identified as floods, but they are not actually floods
4	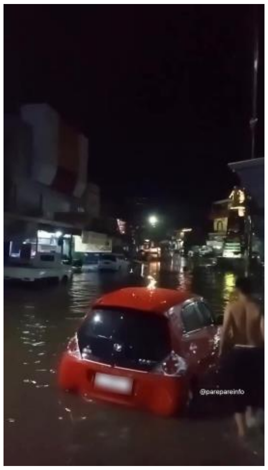	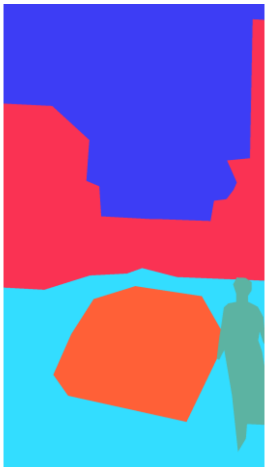	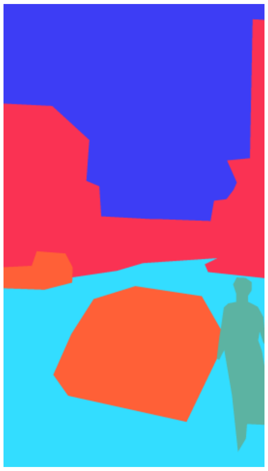	Some items may not be recognized as floods, despite the fact that they are truly floods.
